# Double-Group Particle Swarm Optimization and Its Application in Remote Sensing Image Segmentation

**DOI:** 10.3390/s18051393

**Published:** 2018-05-01

**Authors:** Liang Shen, Xiaotao Huang, Chongyi Fan

**Affiliations:** College of Electronic Science, National University of Defense Technology, Changsha 410000, China; xthuang@nudt.edu.cn (X.H.); chongyifan@nudt.edu.cn (C.F.)

**Keywords:** particle swarm optimization, multilevel thresholding, remote sensing image segmentation, meta-heuristic, swarm intelligence

## Abstract

Particle Swarm Optimization (PSO) is a well-known meta-heuristic. It has been widely used in both research and engineering fields. However, the original PSO generally suffers from premature convergence, especially in multimodal problems. In this paper, we propose a double-group PSO (DG-PSO) algorithm to improve the performance. DG-PSO uses a double-group based evolution framework. The individuals are divided into two groups: an advantaged group and a disadvantaged group. The advantaged group works according to the original PSO, while two new strategies are developed for the disadvantaged group. The proposed algorithm is firstly evaluated by comparing it with the other five popular PSO variants and two state-of-the-art meta-heuristics on various benchmark functions. The results demonstrate that DG-PSO shows a remarkable performance in terms of accuracy and stability. Then, we apply DG-PSO to multilevel thresholding for remote sensing image segmentation. The results show that the proposed algorithm outperforms five other popular algorithms in meta-heuristic-based multilevel thresholding, which verifies the effectiveness of the proposed algorithm.

## 1. Introduction

Particle Swarm Optimization (PSO) is an evolutionary optimization algorithm based on swarm intelligence. It is originally proposed by Kennedy and Eberhart in 1995 [1] and is known for its effectiveness and simplicity. It has been proved to be outstanding in solving many complex optimization problems such as power systems [2], neural network training [3], global path planning [4], and feature selection [5].

However, PSO also suffers from two limitations. One is that the original PSO tends to converge to the local optima when applied to complex problems. On the other hand, the convergence speed of the original PSO and most of its variants is slow, especially on high-dimensional problems [6]. Therefore, accelerating the convergence speed and avoiding the local optima convergence have become the two most important and appealing goals in particle swarm optimization studies [7,8]. Specifically, the studies can be classified into three strategies: parameter selection strategy, topology strategy and learning strategy.

The parameter selection refers to the optimization of the inertial weight factor, convergence factor, and the acceleration constant. The inertial weight factor is introduced by Shi and Eberhart to improve the update of velocity [9]. Further studies also show that applying linear decreasing [10], nonlinear [11], exponential [12] and Gaussian [13] strategy to optimize the inertia weight can enhance the overall performance. The convergence factor is proposed by Clerc and Kennedy to enhance the final convergence [14]. In addition, detailed studies [15,16,17] show that the acceleration constant takes an important role on convergence performance.

The topology strategy is generally employed to improve exploration and avoid premature convergence. In topology strategy, individuals learn from the neighborhood rather than the whole swarm. Therefore, more information would be shared during the search process, which is useful to improve optimization performance. A number of topologies including ring or circle topology, wheel topology, star topology, pyramid topology, Von Neumann topology and random topology are suggested by Kennedy in [18]. Generally, a large neighborhood is good for simple problems, whereas a small neighborhood is helpful for avoiding premature convergence on complex problems [19]. Reference [20] studied the topology extensively, which provides a useful guide of topology selection. It points out that an optimal topology is both problem-specific and computational-budget-dependent and two formulas have been introduced to estimate optimal topology parameters based on numerical experiments. 

In the original PSO, all individuals keep learning from the global best solution and their individual best experience in the whole search process. This may lead to premature convergence [21]. To overcome the problem, some novel learning strategies have been developed in recent years. A comprehensive learning strategy is developed to improve the performance on complex multimodal functions in [22]. Reference [23] introduces a cooperative approach to solve high-dimensional optimization problems with multiple swarms. A cooperatively coevolving strategy is proposed in [24] to further improve the performance. Sun et al. introduce a global guaranteed convergence optimizer called quantum behaved particle swarm optimization, which improves the performance by increasing the population diversity [25]. A variant with double learning patterns is developed in [26], which employs the master swarm and the slave swarm with different learning patterns to achieve a trade-off between the convergence speed and the swarm diversity.

However, the three strategies above still face the following shortcomings. In parameter selection, some strategies do improve the overall performance in many cases, but the effect is limited [19], and it is hard to obtain an optimal parameter for all cases. In topology strategy and learning strategy, although the exploration is improved to avoid premature convergence, the convergence speed is reduced at the same time.

In this paper, we design a double-group particle swarm optimization (DG-PSO) to improve the performance. The whole population is divided into two groups: an advantaged group and a disadvantaged group. The modification is focused on the disadvantaged group. A novel learning strategy is developed based on the comprehensive learning strategy and the self-pollination strategy in another popular metaheuristic called Flower Pollination Algorithm (FPA). In addition, a diversity enhancing strategy is also designed to avoid premature convergence. Compared with those published works, the main contribution in this paper is that a novel variant called DG-PSO is proposed which shows remarkable performance compared with five other popular variants and two meta-heuristics. Two new ideas are developed in DG-PSO: a learning strategy, which combines the comprehensive learning strategy [22] and the self-pollination strategy [27], and a diversity enhancing strategy, which adds disturbance to the individuals in the disadvantaged group to avoid premature convergence in multimodal problem. In addition, we also apply the algorithm to multilevel thresholding for image segmentation, which verifies the effectiveness of DG-PSO and provides a good choice of the metaheuristic algorithm to implement multilevel thresholding. The rest of the paper is organized as follows: Section 2 reviews the original PSO and some related works. The strategies and framework of the proposed algorithm are presented in detail in Section 3, followed by the experiments in Section 4. Then, the further application on multilevel thresholding for image segmentation is shown in Section 5.

## 2. Background

In this section, firstly, we outline the original PSO. Then, two basic works for our algorithm including the comprehensive learning strategy and the self-pollination strategy in FPA are introduced, respectively.

### 2.1. Particle Swarm Optimization

Similar to other meta-heuristics, PSO is based on swarm intelligence. The swarm is composed of a set of particles i∈[1,2,…n]. A particle moves in the search space with a velocity. The position and velocity of the particle are dynamically adjusted according to its own and its companion’s historical experience. Each particle’s position is associated with a candidate solution to the problem, and better solutions are obtained via evolution. The performance of a solution is judged by a given fitness function (e.g., smaller fitness function values indicate better solutions for the minimization problem). For a *D*-dimensional problem, there are four main vectors:

1. The velocity $\left(v_{i}=[v_{i}^{1},v_{i}^{2}\ldots v_{i}^{D}]\right)$: $v_{i}$ denotes the moving speed and direction of the particle i.
2. Position $\left(x_{i}=[x_{i}^{1},x_{i}^{2}\ldots x_{i}^{D}]\right)$: $x_{i}$ is the current position of particle i. It is updated using its velocity $v_{i}$. It can be regarded as a candidate solution.
3. Previous best position $\left(pbest_{i}=[pbest_{i}^{1},pbest_{i}^{2}\ldots pbest_{i}^{D}]\right)$: $Pbest_{i}$ represents the historical best position of particle i. It is updated using the best position that particle i has ever found.
4. Global best position $\left(gbest=[gbest^{1},gbest^{2}\ldots gbest^{D}]\right)$: gbest is the best position the swarm has ever found. It is updated with the best pbest in each generation. The final gbest corresponds to the final solution of the whole algorithm.

Note that each of the solutions or candidate solutions represent a set of to-be-optimized parameters, where D is the number of parameters. In the original PSO, the position and velocity are updated by learning from the current global best solution and its previous best solution according to Equations (1) and (2):

(1) $$v_{i}^{d}(t+1)=w\times v_{i}^{d}(t)+c_{1}r_{1}\times \left(pbest_{i}^{d}-x_{i}^{d}(t)\right)+c_{2}r_{2}\times (gbest^{d}-x_{i}^{d}(t)),$$

(2) $$x_{i}^{d}(t+1)=x_{i}^{d}(t)+v_{i}^{d}(t+1),$$

where i∈[1,2,…n] is the ith particle; $c_{1}$, $c_{2}$ are called acceleration constants; $r_{1}$, $r_{2}$ are two uniformly distributed random number within [1,0]; and w is the inertial weight factor.

### 2.2. Comprehensive Learning Strategy

In the strategy of original PSO, the particles only learn from the global best solution, while its personal best solution may lead to premature convergence. As a consequence, the Comprehensive Learning PSO (CLPSO) is developed to improve the learning strategy [22]. It employs a comprehensive learning strategy, which allows each particle to learn from many particles. Specifically, each dimension of the particle in CLPSO learns from a random particle in the swarm as Equation (3) shows:

(3) $$v_{i}^{d}(t+1)=w\times v_{i}^{d}(t)+c\times r_{i}^{d}\times \left(pbest_{f(i,d)}^{d}-x_{i}^{d}(t)\right),$$

where f(i,d) is the function to define which particle’s pbest we should choose (for the ith particle to follow and learn from). Specifically, for each dimension d of particle i, a random number is generated. If the number is larger than a certain threshold, then the corresponding dimension will learn from its own pbest. Otherwise, f(i,d) works as follows:

1. Randomly choose two particles out of the whole population excluding the particle whose velocity is updated;
2. Compare the fitness of the two particles’ pbest and choose the better one;
3. Use the winner’s pbest as the exemplar for the dth dimension of the particle to learn from using Equation (3).

Specially, if all the winners are the pbest of their own ($pbest_{i}$), it will randomly choose one dimension from the pbest of another particle to learn from. The framework of CLPSO is very similar to the original PSO, and it has been well tested that CLPSO is effective in optimizing benchmark functions and real-world problems [22,28,29,30,31].

### 2.3. Self-Pollination Strategy in the Flower Pollination Algorithm

Flower pollination algorithm is a popular nature inspired meta-heuristic in [27]. It has been widely used in many fields such as sizing optimization of truss structures [27], economic load dispatch problem in power systems [32], Sudoku Puzzles [33] and feature selection [34] since being published in 2012.

As a swarm-based metaheuristic algorithm, each individual i in the swarm is called a pollen individual. Each pollen individual is associated with a candidate solution $\left(sol_{i}=[sol_{i}^{1},sol_{i}^{2}\ldots sol_{i}^{D}]\right)$ in the search space. FPA searches using the global and local search techniques, where the local search simulates the self-pollination process. The self-pollination strategy is one of the basic ideas in FPA (the other one is cross-pollination). Self-pollination occurs when there are no pollen vectors (Pollen vectors, or called pollinators, can be very diverse. It is estimate there are at least 200,000 variety of pollen vectors such as insects, bats and birds [27]) such as wind or insects or when the pollen individuals are pollinated within the same plant. Such self-pollination behaviors are concluded in the following two rules below:

1. Self-pollination corresponds to the local pollination.
2. Pollinators can develop flower constancy, which is regarded as a reproduction probability that is proportional to the similarity of two flowers involved.

Based on the two rules above, the self-pollination strategy is drawn as Equation (4) shows. Different from PSO, $sol_{i}$ is the only vector that associates with each pollen individual. $sol_{i}$ not only represents the position of the pollen individual i, but also plays the role of the best solution this individual has ever found (to understand what is the “sol”, we can refer to the solutions in PSO such as the position x and the previous best solution pbest). It generates the new solutions by using the previous one and two other solutions chosen randomly from the population:

(4) $$sol_{i}^{d}=sol_{i}^{d}+\varepsilon (sol_{r1}^{d}-sol_{r2}^{d}),$$

where $sol_{r1}$ and $sol_{r2}$ are two random solutions in the current generation, which mimics the flower constancy in a limited neighborhood. ε is a uniformly distributed random number within [0,1] used to implement a local random walk. As rule 1 indicates, the self-pollination is considered as local pollination, which often occurs in a limited neighborhood of the particle itself. It can be regarded as the local search around the current position of the pollen individual.

## 3. The Proposed Algorithm

**Figure 1:**
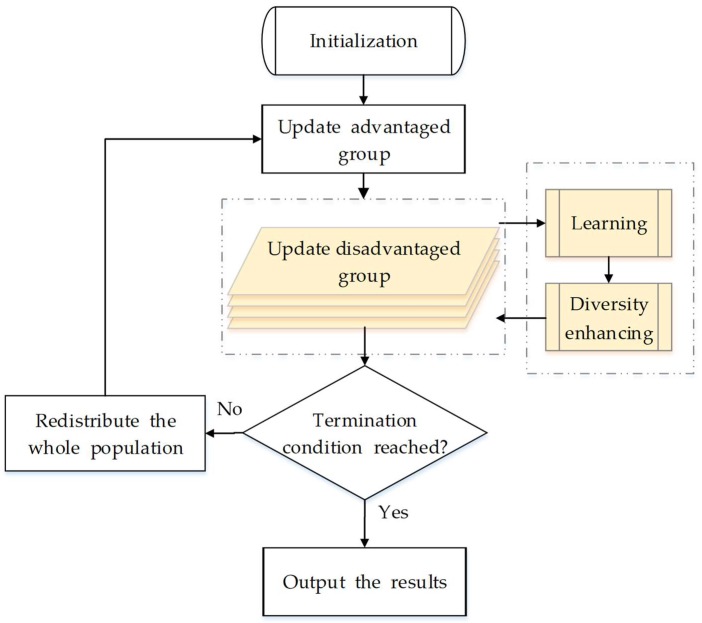
The overall framework of the proposed algorithm.

**Figure 2:**
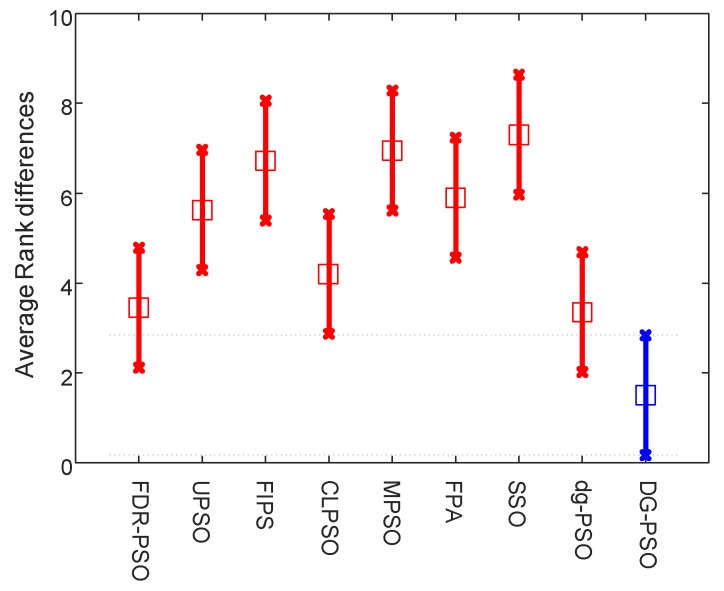
Friedman test of 30D problems.

In this section, we describe the proposed algorithm. Figure 1 shows the overall flowchart, where the process colored by yellow is the core idea of our algorithm. Different from the original PSO, we separate all particles into two groups in DG-PSO: an advantaged group (with the population of $x_{1},x_{2}\ldots x_{m}$) and a disadvantaged group (with the population of $x_{m+1},x_{m+2}\ldots x_{n}$, where n>m). The advantaged group evolves according to the same theory as the original PSO (Equations (1) and (2)), while the disadvantaged group is updated with two novel strategies: a learning strategy and a diversity enhancing strategy. We focus on the explanation of how the disadvantaged group works. As shown in Figure 2, the two new strategies work as two sequential processing stages in the update of the disadvantaged group, which will be discussed carefully in the following two subsections. In addition, the detailed steps and the whole framework of the proposed method are given in Section 3.3. Finally, we discuss and compare the proposed algorithm with other related works in Section 3.4.

### 3.1. The Learning Strategy

The learning strategy is based on the self-pollination strategy introduced in Section 2. We firstly employ the previous best solution pbest to be the solution “sol” in Equation (4) (rather than the position x, this is because pbest represent the best historical experience of each particle, which is more worthy to learn from compared with the position x). Then, it becomes Equation (5) for the particle i:

(5) $$x_{i}^{d}=pbest_{i}^{d}+\varepsilon \times (pbest_{r1}^{d}-pbest_{r2}^{d}),$$

where i=m+1,…,n denotes the particles in the disadvantaged group; $pbest_{r1}$ and $pbest_{r2}$ are two solutions chosen randomly from the pbest of the whole population (Specifically, r1 and r2 are two random integers chosen from sequence 1,2,…n (r1≠r2). These two parameters keep the same for all dimensions when updating a particle i. In addition, they are regenerated for different particles.). ε represents the scaling factor to perform a random walk satisfying a uniform distributed within [0,1]. Similar to the original self-pollination strategy, Equation (5) can be considered as the local search around the solution (position) $pbest_{i}$.

On the other hand, as the comprehensive learning strategy generally defines a more suitable solution for the particles to learn from, we additionally replaced the $pbest_{i}^{d}$ in Equation (5) with $pbest_{f(i,d)}^{d}$ given in Equation (6), where f(i,d)∈[1,2…m] is the strategy to identify a particle’s pbest for the dth dimension of particle i to learn from:

(6) $$x_{i}^{d}=pbest_{f(i,d)}^{d}+\varepsilon \times (pbest_{r1}^{d}-pbest_{r2}^{d}),$$

f(i,d) works according to the comprehensive learning strategy. For the dth dimension of particle i, the specific procedure to identify the $pbest_{f(i,d)}^{d}$ is shown as follows:

1. Randomly choose two particles out of the advantaged group;
2. Compare the fitness of the two particles’ pbest and choose the better one;
3. Use the dth dimension of the winner’s pbest as the $pbest_{f(i,d)}^{d}$ for the corresponding dimension of the ith particle to learn from.

Then, a new position is generated using Equation (6) for the particle i in the disadvantaged group to update. Using (6), the particles in the disadvantaged group can learn from the information derived from different particles’ historical best position. The strategy is different from the original self-pollination because we perform local search around the new generated position $pbest_{f(i,d)}^{d}$ rather than the particle itself. The reason is that always searching the area around the position itself may reduce the search efficiency because some particles may be located in the low-promising area. In contrast, making more use of the good information from the advantaged group (using the comprehensive learning strategy) is inductive to the search efficiency.

### 3.2. The Diversity Enhancing Strategy

PSO often suffers from premature convergence, especially when optimizing the multimodal problem. It is because the original PSO algorithm only employs an attraction phase Equation (1), in which all particles in the swarm move quickly to the same area and the diversity decreases quickly [35]. This generally leads to converging to the local optima due to the loss of diversity [22]. In such case, improving diversity becomes an important issue in PSO research [22,36]. As diversity is lost due to particles getting clustered together [37], adding disturbance to the particles is helpful for them to escape from the local optimal and enhance diversity. Therefore, we developed a strategy to push the particle away from their current position by adding disturbance given in Equation (7):

(7) $$x_{i,d}=x_{i,d}+rand_{1}\times s,$$

where s is the scaling factor that controls the intensity of the disturbance. As shown in Equation (8), it is identified using the whole search range of the corresponding dimension (which denotes the strong disturbance) or the Euclidean distance of the two pbest chosen in the learning strategy (which denotes a relatively weak disturbance). The strong disturbance is designed for the case that the particle falls $rand_{1}$ into a large-area local optimum. Therefore, a big jump is needed to escape. The weak disturbance is designed for the case that the particle is close to the global optimum. In such case, a small random walk is more helpful to approaching the optimum.

(8) $$s=\left\{ \begin{array}{ll} \left|{Ub}_{d}-{Lb}_{d}\right|, & {rand}_{2}<0.5,\\ \|{pbest}_{r1}-{pbest}_{r2}\|, & otherwise, \end{array}\right.$$

where, $rand_{1}$ and $rand_{2}$ are two random number uniformly generated within [0,1] and Ub and Lb represents the upper and lower bounds of the search space.

Specifically, the strategy works as follows. For each dimension of particle i, we generate a random number within [0,1]. If the number is smaller than the given threshold P, the diversity of the corresponding dimension will be enhanced by adding a random disturbance using (7) and (8). With the disturbance, the particles are more capable to escape from the local optimal and avoid premature convergence.

### 3.3. The Framework

Algorithm 1 shows the detailed steps of updating the disadvantaged group, which is the core of our modification. Apart from Algorithm 1, another minor modification in the proposed algorithm is that all particles in the two groups should be redistributed according to their fitness at the end of each generation. m particles with better fitness (for minimization problem, “better” means “smaller”) are distributed to the advantaged group, whereas others are distributed to the disadvantaged group. The overall framework and the detailed steps are shown in Figure 1 and Algorithm 2, respectively, where *MaxFEs* is the maximum number of function evaluations that represent the maximum computation cost.

**Table:** 

**Algorithm 1.** The Steps for Updating the Disadvantaged Group	
1	**For** i=m+1:n
2	Randomly choose two pbest:$pbest_{r2}$ and $pbest_{r2}$ out of the whole population;
3	/* Learning stage */
4	**For** d=1:D
5 ^1^	Generate two different integers a and b within [1,2…*m*];
6 ^2^	**If** $fpbest_{a}<fpbest_{b}$
7	$pbest_{f(i,d)}^{d}=pbest_{a}^{d}$;
8	**Else**
9	$pbest_{f(i,d)}^{d}=pbest_{b}^{d}$;
10	**End**
11	$x_{i}^{d}=pbest_{f(i,d)}^{d}+\varepsilon \times (pbest_{r1}^{d}-pbest_{r2}^{d})$;
12	**End**
13	/* Diversity Enhancing stage */
14	**For** j=1:D
15	**If** rand<p
16	Draw a scaling factor using Equation (8);
17	Add disturbance for the current dimension using Equation (7);
18	**End**
19	**End**
20	**End**

^1^ This step aims to choose two *pbest* from the advantaged group; ^2^ *fpbest* stands for the fitness value of the *pbest*, which has been recorded before.

**Algorithm 2.** The Steps of the Proposed Algorithm1Randomly initialize *n* particles;2*m* particles with better fitness value for the advantaged group; others for the disadvantaged; 3
**While**
fes<MaxFEs
4         **For**
i=1:m5               Update the particle i in the advantaged group using Equations (1) and (2);6        **End**7        Evaluate the fitness of the advantaged group; 8         Update *pbest* and record the corresponding fitness as *fpbest*.9        Update the disadvantaged group using Algorithm I;10        Evaluate the fitness of the disadvantaged group;11         Update *pbest* and record the corresponding fitness as *fpbest*.12        fes=fes+n;13         Redistribute the whole population;14
**End**


### 3.4. Discussion and Comparison of the Proposed Algorithm with Other Related Works

As mentioned above, we combined the current existing comprehensive learning strategy with the self-pollination strategy in FPA. Specifically, we firstly applied the self-pollination strategy to PSO. Then, the comprehensive learning strategy is used to identify an exemplar for the particles in the disadvantaged group to learn from. Note that we choose the exemplar in the advantaged group rather than in the whole swarm. This strategy aims to improve the learning efficiency of the disadvantaged group. Obviously, such strategy is different from CLPSO (because CLPSO uses the comprehensive learning to modify the learning strategy of the original PSO as introduced in Section 2, whereas we proposed a new learning strategy).

Based on the analysis above, CLPSO, FPA would be used to compare with the proposed one. In addition, since we also developed a diversity enhancing strategy to further improve the performance, it is also necessary to evaluate its effectiveness. We firstly define:

1. dg-PSO: the proposed algorithm that only employs the learning strategy;
2. DG-PSO: the proposed algorithm that employs both the learning strategy and the diversity enhancing strategy.

Then, the effectiveness of the diversity enhancing strategy can be evaluated by comparing the performance of dg-PSO with DG-PSO.

## 4. Experiments on Benchmark Functions

In this section, we first describe the 20 benchmark functions used for performance evaluation. Then, the algorithms and the necessary parameters for comparison are introduced. Finally, the results are shown and discussed in detail.

### 4.1. The Benchmark Functions

The 20 benchmark functions employed in the experiments are presented in Table 1. All the functions are the minimization problem, which is defined according to [38,39] in the search space [−100,100]. The functions can be categorized into four classes, namely (1) basic problems; (2) rotated problems; (3) shifted problems; and (4) complex problems. The basic problems include not only the basic unimodal and multimodal problems, but also a noisy problem (F4), an expanded (F8) and an expanded hybrid problem (F9). The rotated problems are designed to overcome the drawback in the basic functions that the variables are separable and the local optima are regularly distributed. In these rotated problems, the original variable x is rotated by left multiplying the orthogonal matrix M, i.e.,y=M×x. Shifted problems are designed to overcome two other problems (in basic functions) including: each dimension value of the global optimum is always the same, and the global optimum is usually located at the centre of the search space. In addition, the complex problems include both rotation and shift.

### 4.2. Algorithms and Parameters

Table 2 shows the five PSO variants and two other popular meta-heuristics used in the comparison. These algorithms include not only the algorithms we mentioned before (CLPSO, FPA), but also some other state-of-the-art algorithms, which are chosen according to the three strategies introduced in Section 1. We give a brief description of them here. First, Modified PSO (MPSO) [36] uses parameter selection based strategy, of which the population size and inertial weight are adaptively adjusted within the search process. Second, Unified PSO (UPSO) [40] and Fully Informed PSO (FIPS) [41] are two neighbourhood topology strategy based variants. UPSO represents the unified PSO, which is a combination of the original PSO and the topology strategy based PSO. FIPS means the fully informed PSO, which employs the fully informed neighbourhood topology. Finally, Fitness-distance-Ratio PSO (FDR-PSO) [42] and CLPSO [22] are chosen from learning strategy based variants, where FDR-PSO employs a fitness-distance-ratio strategy to identify a “fittest-and-closest” particle to modify the learning strategy. In addition, another novel meta-heuristic called Social Spider Optimization (SSO) [43] is also chosen to give the comparison as comprehensive as possible. In addition, DG-PSO and dg-PSO are the proposed algorithms, where only DG-PSO has diversity enhancing strategy.

The parameters of the involved algorithm are set as follows. For dg-PSO and DG-PSO, the population size of the advantaged group and the disadvantage group are set to 30 and 25 respectively; the possibility p of diversity enhancing is set to 1/D. The population size for other PSO variants are set to 40 [44], except MPSO, which employs the adaptive population strategy (initial value, minimum and maximum are 5, 5 and 40, respectively) [36]. Other parameters are listed in Table 2. We performed the evaluation in both 30 dimensions with $MaxFEs=4\times 10^{5}$ [45] and 50 dimensions with $MaxFEs=7\times 10^{5}$. Thirty runs are conducted for each function, and the mean fitness error and the corresponding deviation are calculated (the error is defined by the difference between the fitness function value and the minimum, i.e., $Error=Fitness-F_{\min}$). All the experiments are carried out using MATLAB 2016 on the same machine with an Intel I5-4590 CPU @ 3.3 GHz processor (Intel, Santa Clara, CA, USA), 4.00 GB memory, and Windows 7 Professional operating system (Microsoft, Redmond, WA, USA).

### 4.3. Results and Discussion

The mean fitness error values and the corresponding standard deviation are shown in Table 3 and Table 4, respectively, where “Mean” represents the mean fitness error of which the best one in each case is shown in bold; “Std” means the standard deviation. We perform the Wilcoxon Signed Rank test to give a rigorous comparison, in which the significance level is set as 0.05. The results are represented by “C” in the tables, where the three kinds of symbols indicate the performance of DG-PSO: “+” means DG-PSO is relatively better, “=“ means insignificant and “-” means DG-PSO is relatively worse. We make a sum of the comparison results and showed the final results in the form of “W/T/L” in the bottom of each table, where “W/T/L” means the number of problems DG-PSO win, tie and lose respectively compared with the corresponding algorithm.

We firstly compare DG-PSO with other published algorithms. According to the statistical results in Table 3 (30D), DG-PSO showed better or close performance in all functions when comparing with CLPSO (W/T/L = 18/2/0) and FPA (W/T/L = 19/1/0). In addition, in the comparison with other PSO variants, FDR-PSO (W/T/L = 18/1/1) seems to be the most competitive one to the proposed algorithm (except dg-PSO), however, it only wins in one case. In addition, DG-PSO even wins in all 20 comparisons compared with the recently published meta-heuristic SSO. Similar results are obtained in 50D where DG-PSO still shows great advantages over all other algorithms. The most competitive algorithm to the proposed algorithm is FDR-PSO (W/T/L = 17/2/1) (except dg-PSO), but, obviously, the results still show the superiority of our algorithm. Then, comparing DG-PSO with dg-PSO, the results are W/T/L = 13/1/6 in both 30D and 50D. Specifically, DG-PSO shows much better performance on multimodal problems such as F3, F5, F6, F8, F9, F14, F15, F16, F19 and F20. However, by comparing DG-PSO with dg-PSO, we can find that the diversity enhancing also brings significant inefficiency to DG-PSO on unimodal problems (F1, F2, F4, F10, F17 and F18). This is mainly because the diversity enhancing strategy weakens the exploitation.

**Figure 3:**
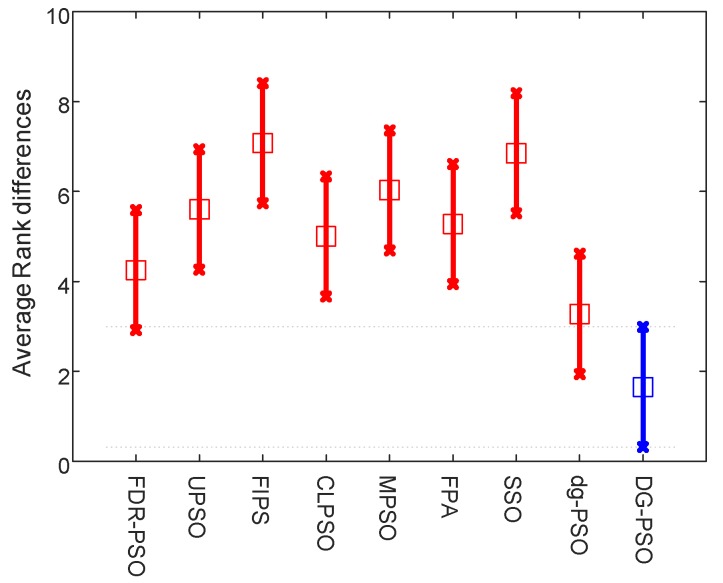
Friedman test of 50D problems.

To rank the algorithms clearly, the Friedman test is used to compare the involved algorithms using all the data of mean fitness error values on the 20 problems. The Friedman test is the best-known procedure for testing the differences between more than two related samples [48], which can detect significant differences between the behavior of two or more algorithms. We conduct two tests that rank the algorithms on the 30D and 50D, respectively. The significance level is set to 0.05. Table 5 presents the numerical rankings obtained by the test. In addition, the corresponding graphical ranking results are shown in Figure 2 and Figure 3, where the center square indicates the average rank of the corresponding algorithm and the line denotes the confidence intervals. Smaller ranks mean better performance and, when there is no overlap on the intervals of any two algorithms, they are significantly different. The results in these two figures clearly demonstrate that the proposed algorithm outperforms all other algorithms including dg-PSO, CLPSO and FPA in both 30D and 50D.

**Figure 4:**
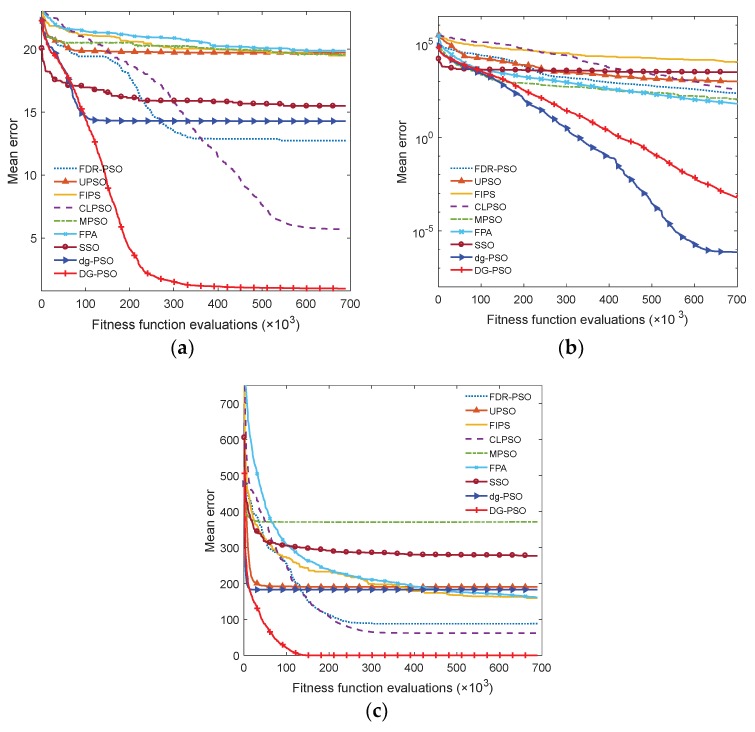
Convergence performance. (**a**) F8, multimodal; (**b**) F10, rotated unimodal; (**c**) F14, shifted multimodal.

**Figure 5:**
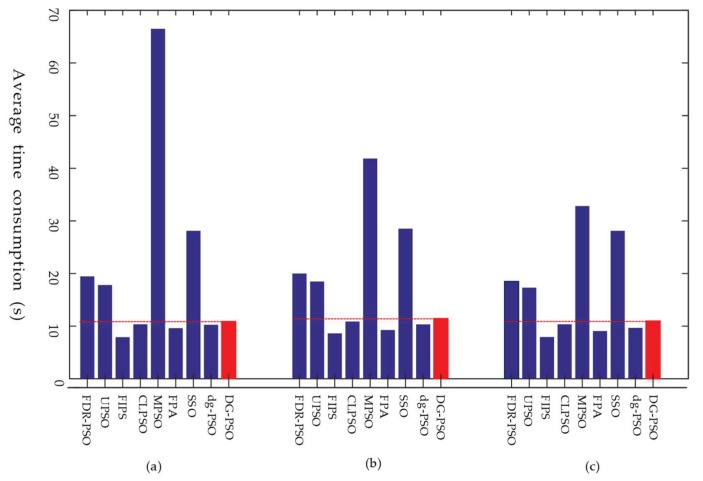
Average time consumption. (**a**) F8, multimodal; (**b**) F10, rotated unimodal; (**c**) F14, shifted multimodal.

For further evaluation, the convergence performance and average time consumption are also compared in Figure 4 and Figure 5, respectively. The results of F8, F10, and F14 in 50-*D* are given to exemplify the performance. From Figure 4, we observe that DG-PSO has outstanding performance on the multimodal problems (F8 and F14), while dg-PSO obtained the best result in unimodal function F10. From Figure 5, it can be found that DG-PSO consumes slightly more than the two related algorithms: CLPSO and FPA. However, the time consumption of DG-PSO is still acceptable when compared with the other algorithms such as FDR-PSO, UPSO, MPSO and SSO.

## 5. DG-PSO Based Remote Sensing Image Segmentation

Image segmentation is a fundamental task in remote sensing applications [49], such as change detection and object-based classification. It is used with the expectation that it will divide the image into semantically significant regions, or objects, to be recognized by further processing steps [50]. This work attracts a lot of researchers in the past decade but is still an intractable problem [51]. In terms of all the existing segmentation methods, one of the most popular segmentation techniques is thresholding due to its simplicity, robustness and accuracy [52].

The thresholding methods can be divided into two categories: the bi-level thresholding and multilevel thresholding. If the object in an image is separated from the background using a single threshold value, it is called the bi-level thresholding. In contrast, the multilevel thresholding means that the given image are classified into several different regions according to multiple thresholds. In remote sensing image segmentation, bi-level thresholding does not give appropriate performance, and there are strong requirements of multilevel thresholding [53]. Therefore, numerous studies have been reported [47,53,54,55,56,57,58] in multilevel thresholding.

The most popular way [53,54,55,56,57,58,59,60,61] to search the optimal thresholds is to maximize some discriminating criteria (fitness function). The traditional method searches the optimal thresholds using exhaustive search strategies, which lead to high computation costs. In recent years, meta-heuristics based methods gained the attention of researchers because of the high computation inefficiency. Quantities of algorithms have been introduced to this area such as PSO [36], Differential Evolution (DE) [62], Artificial Bee Colony (ABC) [59,63,64], Wind Driven Optimization (WDO) [56], Cuckoo Search (CS) [65] and SSO [47]. However, the remote sensing images are very difficult to segment accurately due to multimodality of the histograms [53]. Therefore, improving the performance of the metaheuristic algorithms is necessary for the remote sensing image segmentation.

In this section, we applied the proposed algorithm to multilevel thresholding for optical remote sensing image segmentation. We first describe the problem. Then, the experimental setup is introduced carefully in Section 5.2. Finally, the results and analysis are given in detail.

### 5.1. Problem Definition

This subsection deals with the problem definition of multilevel thresholding problem. As we mention above, multilevel thresholding methods generally search the optimal thresholds by maximizing some criteria. In the literature, Otsu’s criterion [66] has been widely employed [36,67,68]. It generally provides image segmentation with satisfactory results [69] and is known for its simplicity and effectivity with respect to uniformity and shape measures and can usually obtain optimal global threshold value [58].

Let l∈[0,1…L−1] be the gray level of a given an image I, where L is the total gray levels, the problem is then defined as follows. Firstly, the image histogram is calculated and normalized, which is denoted by $P_{l}$, l=0,1,…L−1. For the (D+1)−class thresholding problem, there are D thresholds $k_{d}$, (d=1,2,…D) that segment the image into D+1 classes. Assume that $k_{0}(k_{0}=0)$ and $k_{D+1}(k_{D+1}=L)$ denote the upper and lower bound. Then, the thresholds can be sorted with $k_{0}<k_{1}<\ldots <k_{d}<\ldots k_{D+1}$, and the problem is defined using (9):

(9) $$(k_{1}^{*},k_{2}^{*}\ldots k_{D}^{*})=\underset{k_{0}<k_{1}<\ldots <k_{d}<\ldots k_{D+1}}{\arg}\max\left\{F\left(k_{1},k_{2}\ldots k_{D}\right)\right\},$$

where $F=\sum_{d=0}^{D}\omega_{d}{\left(\mu_{d}-\mu_{T}\right)}^{2}$, $\mu_{d}=\sum_{l=k_{d}}^{k_{d+1}}\frac{l\cdot P_{l}}{\omega_{d}}$. Here, $\omega_{d}=\sum_{l=k_{d}}^{k_{d+1}}P_{l}$ is the probability of the occurrence of the *d*th class. $\mu_{T}=\sum_{l=1}^{L}l\cdot P_{l}$ is the total mean intensity of the original image.

### 5.2. Experimental Setup

To demonstrate the superiority of the proposed method, five popular meta-heuristic algorithms in multilevel thresholding including DE, ABC, CS, MPSO, SSO are chosen to compare with the proposed algorithm. All of these algorithms are demonstrated to have good performance in multilevel thresholding in the corresponding reference in Table 6. Specifically, ABC performs better than PSO when the level of thresholds is higher than two in [59]. Reference [53] demonstrates that CS showed remarkable performances in multilevel thresholding problems and could outperform the other known algorithms, such as DE, PSO, WDO and ABC. MPSO shows better performance than Genetic Algorithm (GA) and the original PSO [36]. SSO is applied to multilevel thresholding in [47] and it clearly outperforms PSO, BAT algorithm and FPA in [47]. The parameters of these algorithms are set according to the corresponding work shown in Table 6. The parameters of our proposed algorithm are the same as that in Section 4.

**Figure 6:**
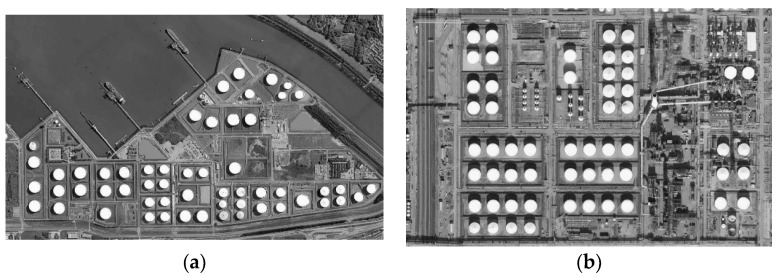

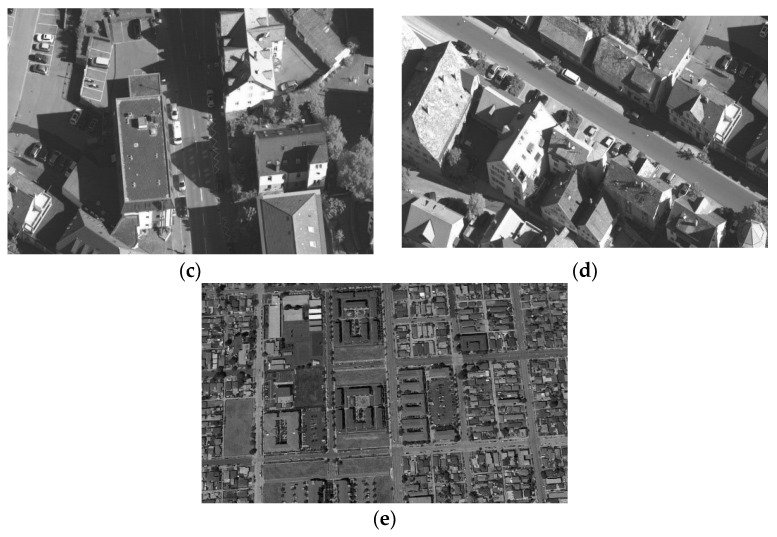
Images used in the experiments. (**a**) Image a; (**b**) Image b; (**c**) Image c; (**d**) Image d; (**e**) Image e.

All populations are uniformly randomly initialized. Thirty independent runs are carried out for each algorithm on each image on 2, 3, 4, 5, 7, 9, 15 and 20 thresholds [68,69], respectively. All algorithms are conducted with the same maximum function evaluation: MaxFEs=3000*D in identical search space: [0,256). All methods are adapted for integer optimization problems using the rounding method. Specifically, the search space is defined as [0,256) for 8-bit gray-scale images, and the integer is obtained by rounding down (e.g., 255.6 is rounded to 255). Figure 6 shows the test images (These images are taken from a very-high-resolution remote sensing image dataset constructed by Gong Cheng et al. from Northwestern Polytechnical University [70].

### 5.3. Results and Discussion

**Figure 7:**
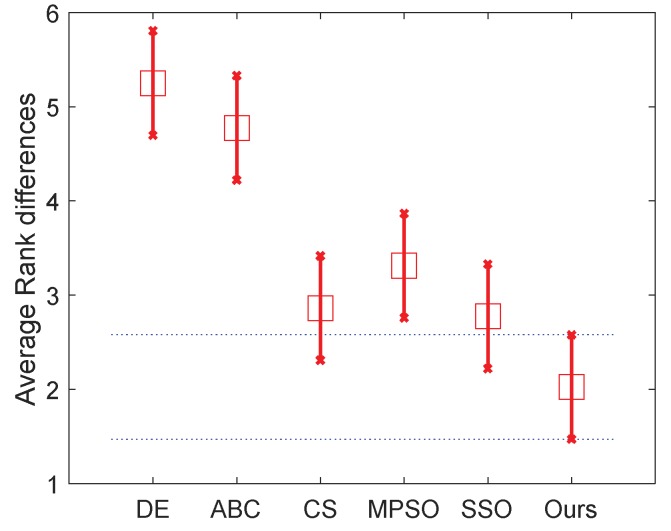
Friedman test of the normal level.

**Figure 8:**
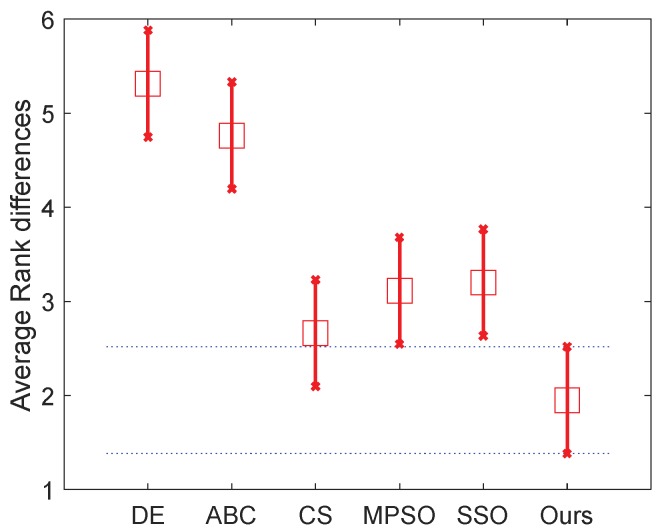
Friedman test of the high level.

In detail, the mean fitness and the corresponding standard deviation are given in Table 7, where the best one in each case of the mean fitness is shown in bold. It is easy to find that our algorithm obtains the best results in all cases in terms of the mean fitness, except the case of 7-level thresholding (*D* = 7) of image C. To evaluate the effectiveness of our algorithm’s improvement over other ones, the involved algorithms are also ranked with the Friedman test. We conduct two tests that ranked the algorithms on the normal (*D* = 2, 3, 4 and 5) level and high level (The high level thresholding is popularly employed in multilevel thresholding [68,69]) (*D* = 7, 9, 15 and 20), respectively. Therefore, 40 variables (i×t×m=40) are used in each comparison in each test, where i=5 is the number of images, t=4 denoted the number of levels, and m=2 denoted the number of used measures including the meant fitness and the corresponding standard deviation. The significance level is set to 0.05. Table 8 and the two figures (Figure 7 and Figure 8) present the numerical rankings and graphical results obtained by the test, where better performance is denoted by smaller ranks.

**Figure 9:**
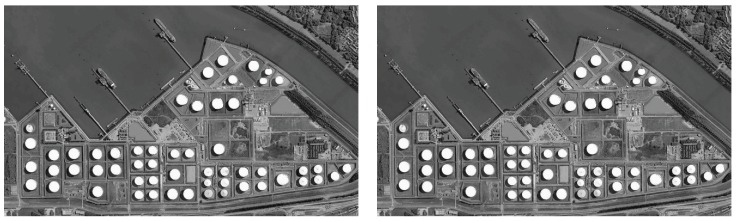

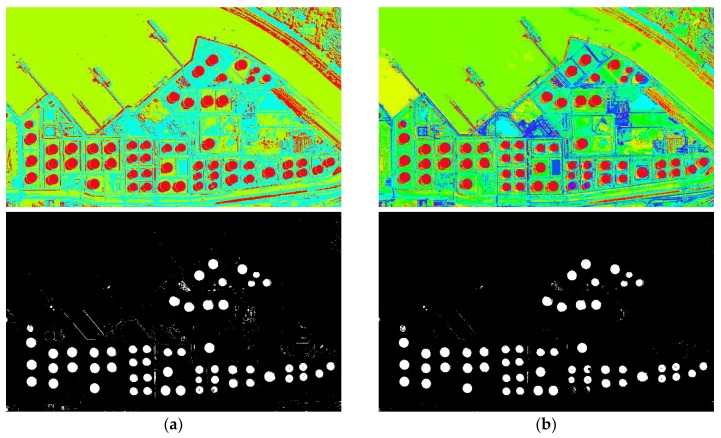
The segmentation results of the Image a. (**a**) 3-level thresholding; (**b**) 9-level thresholding.

**Figure 10:**
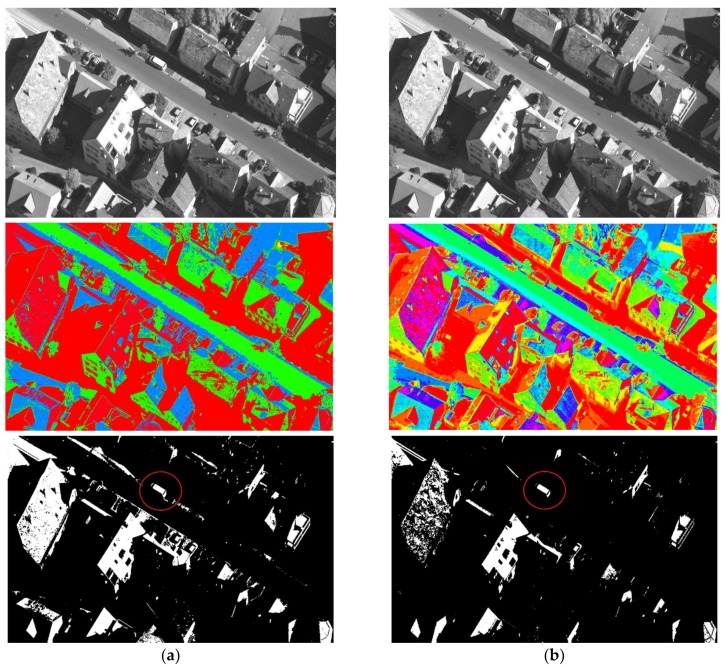
The segmentation results of the Image d. (**a**) 3-level thresholding; (**b**) 9-level thresholding, which separated the target better.

From the results of normal level thresholding shown in Figure 7, the proposed algorithm significantly outperforms DE, ABC and MPSO, and also showed an advantage over the other two algorithms. It can be observed from Figure 8 that the proposed algorithm ranks even better in high level thresholding, which showed a significant difference from all algorithms except CS (our algorithm also ranks better than CS). Figure 9 and Figure 10 show the segmentation results. The pseudo color image shows the whole thresholding results, where each level of the image is represented by the regions with the same color. The binary images show some of the objects separated from the original image, which proved the effectiveness of the segmentation.

In conclusion, the results demonstrated that the proposed algorithm shows remarkable performance in multilevel thresholding when compared with other popular meta-heuristics in this research area.

## 6. Conclusions

This paper proposes a variant of particle swarm optimization called DG-PSO. DG-PSO uses a double-group based evolution framework. The individuals in DG-PSO are divided into two groups according to their fitness values. Two main ideas are introduced in the evolution of the disadvantaged group: a hybrid strategy for learning and a diversity enhancing strategy for avoiding premature convergence. The experimental results on various benchmark functions demonstrate that: although DG-PSO consumes slightly more time than the two related algorithms of the proposed algorithm: CLPSO and FPA; DG-PSO achieves a significant improvement in terms of mean fitness error, the corresponding standard deviation and convergence performance over all contrast algorithms. In addition, we further apply the proposed algorithm to multilevel thresholding for remote sensing image segmentation. The results also show the effectiveness of DG-PSO.

## Figures and Tables

**Table 1:** Description of the benchmark functions.

No.	Name	Definition	$F_{\min}$	Modality
F1	Schwefel 1.2	$F_{1}(x)={\sum_{d=1}^{D}\left(\sum_{j=1}^{d}x_{j}\right)}^{2}$	0	Unimodal
F2	Bent Cigar	$F_{2}(x)=x_{1}^{2}+10^{6}\cdot \sum_{d=2}^{D}x_{d}^{2}$	0	Unimodal
F3	Modified Schwefel	$\begin{array}{l} F_{3}(x)=418.9829\cdot D-\sum_{d=1}^{D}g\left(z_{d}\right),z_{d}=x_{d}+4.209687462275036e^{2}\\ g\left(z_{d}\right)=\left\{ \begin{array}{l} z_{d}\sin\left({\left|z_{d}\right|}^{1/2}\right)\\ \left(500-\mathrm{mod}(z_{d},500)\right)\sin\sqrt{\left|500-\mathrm{mod}(z_{d},500)\right|}-\frac{{\left(z_{d}-500\right)}^{2}}{10000D}\\ \left(\mathrm{mod}(\left|z_{d}\right|,500)-500\right)\sin\sqrt{\left|\mathrm{mod}(\left|z_{d}\right|,500)-500\right|}-\frac{{\left(z_{d}+500\right)}^{2}}{10000D} \end{array}\right. \end{array}$	0	Multimodal
F4	Schwefel 1.2 with Noise	$F_{4}(x)=F_{1}\cdot \left(1+0.4\cdot \left|N(0,1)\right|\right)$	0	Unimodal
F5	Rosenbrock	$F_{5}(x)=\sum_{d=1}^{D-1}\left(100{\left(x_{d}^{2}-x_{d+1}\right)}^{2}+{\left(x_{d}-1\right)}^{2}\right)$	0	Multimodal
F6	Rastrigin	$F_{6}(x)=\sum_{d=1}^{D}\left(x_{d}^{2}-10\cos(2\pi x_{d})+10\right)$	0	Multimodal
F7	Katsuura	$F_{7}(x)=\frac{10}{D^{2}}{\prod_{d=1}^{D}\left(1+d\sum_{j=1}^{32}\frac{2^{i}x_{d}-round\left(2^{i}x_{d}\right)}{2^{i}}\right)}^{\frac{10}{D^{1.2}}}-\frac{10}{D^{1.2}}$	0	Multimodal
F8	Expanded Scaffer F6	$\begin{array}{l} F_{8}(x)=g\left(x_{1},x_{2}\right)+g\left(x_{2},x_{3}\right)+\ldots +g\left(x_{D},x_{1}\right)\\ where\;g\left(x,y\right)=0.5+\frac{\left(\sin^{2}\left(\sqrt{x^{2}+y^{2}}\right)-0.5\right)}{{\left(1+0.001\left(x^{2}+y^{2}\right)\right)}^{2}} \end{array}$	0	Multimodal
F9	Expanded Griewank plus Rosenbrock Function	$\begin{array}{l} F_{9}(x)=g\left(F_{5}\left(x_{1},x_{2}\right)\right)+g\left(F_{5}\left(x_{2},x_{3}\right)\right)+\ldots +g\left(F_{5}\left(x_{D},x_{1}\right)\right)\\ where\;g(y)=\sum_{d=1}^{D}\frac{y_{d}^{2}}{4000}-\prod_{d=1}^{D}\cos\left(\frac{y_{d}}{\sqrt{d}}\right)+1 \end{array}$	0	Multimodal
F10	Rotated Bent Cigar	$F_{10}(x)=F_{2}(z),z=M\times x$	0	Unimodal
F11	Rotated Rosenbrock	$F_{11}(x)=F_{4}(z),z=M\times x$	0	Multimodal
F12	Rotated Expanded Scaffer F6	$F_{12}(x)=F_{8}(z),z=M\times x$	0	Multimodal
F13	Rotated Expanded Griewank plus Rosenbrock	$F_{13}(x)=F_{9}(z),z=M\times x$	0	Multimodal
F14	Shifted Rastrigin	$F_{14}(x)=F_{6}(z)+f_{bias1},z=x-o,f_{bias1}=800$	800	Multimodal
F15	Shifted Expanded Scaffer F6	$F_{15}(x)=F_{8}(z)+f_{bias2},z=x-o,f_{bias2}=1600$	1600	Multimodal
F16	Shifted Expanded Griewank plus Rosenbrock	$F_{16}(x)=F_{9}(z)+f_{bias3},z=x-o,f_{bias3}=1500$	1500	Multimodal
F17	Shifted Rotated Bent Cigar	$F_{18}(x)=F_{2}(x)+f_{bias5},z=M\left(x-o\right),f_{bias5}=200$	200	Unimodal
F18	Shifted Rotated Discus	$\begin{array}{l} F_{17}(x)=g(x)+f_{bias4},z=M\left(x-o\right),f_{bias4}=200\\ g(y)=10^{6}\cdot y_{1}^{2}+\sum_{d=2}^{D}y_{d}^{2} \end{array}$	300	Unimodal
F19	Shifted Rotated Expanded Scaffer F6	$F_{19}(x)=F_{8}(x)+f_{bias6},z=M\left(x-o\right),f_{bias6}=1600$	1600	Multimodal
F20	Shifted Rotated Expanded Griewank plus Rosenbrock	$F_{20}(x)=F_{9}(x)+f_{bias7},z=M\left(x-o\right),f_{bias7}=1500$	1500	Multimodal

**Table 2:** Parameters and references of the involved algorithms.

**PSO Variants**	**Parameters**		**Reference**
FDR-PSO	w=0.9−0.5×g/Max_iter; $c_{1}=c_{2}=2.0$		[42]
UPSO	w=0.7298; $c_{1}=c_{2}=1.49445$		[46]
FIPS	w=0.7298; $c_{1}=c_{2}=1.49445$		[41]
CLPSO	w=0.9−0.5×g/Max_iter; $c_{1}=c_{2}=2.0$		[22]
MPSO	$w_{\min}=0.3$$w_{\max}=0.9$; $c_{1}=c_{2}=2.0$;		[36]
**Other State-of-the-Art Meta-Heuristics**	**Parameters**		**Reference**
FPA	Population size Switch possibility	n=25 p=0.8	[27]
SSO	Population size The threshold	n=50 PF=0.7	[47]
**The Proposed Variants**	**Parameters**		**Reference**
dg-PSO	w=0.9−0.5×g/Max_iter;$c_{1}=c_{2}=2.0$		Ours (without diversity enhancing)
DG-PSO	w=0.9−0.5×g/Max_iter;$c_{1}=c_{2}=2.0$		Ours (with diversity enhancing)

**Table 3:** Statistical results on 30 dimensions.

No.	Item	FDR	UPSO	FIPS	CLPSO	MPSO	FPA	SSO	dg-PSO	DG-PSO
F1	Mean	3.07 × 10^−19^	3.72 × 10^−12^	7.05 × 10^+00^	7.10 × 10^−03^	1.12 × 10^−02^	3.60 × 10^−03^	2.71 × 10^−02^	**1.23 × 10^−39^**	3.45 × 10^−29^
	Std	1.25 × 10^−19^	1.94 × 10^−12^	2.47 × 10^+00^	4.96 × 10^−03^	5.30 × 10^−03^	2.57 × 10^−03^	1.41 × 10^−02^	2.72 × 10^−39^	1.79 × 10^−29^
	C	+	+	+	+	+	+	+	-	/
F2	Mean	5.90 × 10^−206^	9.59 × 10^−176^	4.32 × 10^−27^	6.75 × 10^−78^	9.84 × 10^−15^	3.81 × 10^−05^	8.18 × 10^+02^	**1.28 × 10^−226^**	1.68 × 10^−126^
	Std	0.00 × 10^+00^	0.00 × 10^+00^	1.14 × 10^−27^	3.68 × 10^−78^	6.75 × 10^−15^	3.59 × 10^−05^	1.67 × 10^+02^	0.00 × 10^+00^	1.88 × 10^−126^
	C	-	-	+	+	+	+	+	-	/
F3	Mean	3.00 × 10^+02^	2.48 × 10^+03^	2.58 × 10^+03^	3.79 × 10^+02^	2.43 × 10^+03^	2.49 × 10^+03^	1.50 × 10^+03^	1.47 × 10^+03^	**1.66 × 10^+02^**
	Std	1.71 × 10^+02^	2.75 × 10^+02^	2.28 × 10^+02^	1.35 × 10^+02^	1.73 × 10^+02^	7.24 × 10^+01^	2.60 × 10^+02^	7.50 × 10^+02^	7.96 × 10^+01^
	C	+	+	+	+	+	+	+	+	/
F4	Mean	2.14 × 10^+03^	1.12 × 10^+03^	1.80 × 10^+02^	3.24 × 10^+02^	5.00 × 10^+03^	4.39 × 10^+01^	4.88 × 10^+00^	**7.73 × 10^−03^**	1.37 × 10^−01^
	Std	1.56 × 10^+03^	4.92 × 10^+02^	5.49 × 10^+01^	1.23 × 10^+02^	2.49 × 10^+03^	8.42 × 10^+00^	4.00 × 10^+00^	1.20 × 10^−02^	6.15 × 10^−02^
	C	+	+	+	+	+	+	+	-	/
F5	Mean	4.01 × 10^−04^	1.49 × 10^+00^	2.20 × 10^+01^	2.04 × 10^+01^	3.19 × 10^+01^	6.60 × 10^+00^	1.00 × 10^+01^	1.05 × 10^+00^	**4.22 × 10^−16^**
	Std	1.51 × 10^−04^	1.02 × 10^+00^	2.55 × 10^−01^	6.99 × 10^−01^	1.39 × 10^+01^	4.55 × 10^+00^	8.67 × 10^−01^	1.95 × 10^+00^	2.16 × 10^−16^
	C	+	+	+	+	+	+	+	+	/
F6	Mean	3.04 × 10^+01^	6.93 × 10^+01^	6.11 × 10^+01^	7.76 × 10^+00^	3.42 × 10^+01^	3.67 × 10^+01^	4.31 × 10^+01^	3.08 × 10^+01^	**0.00 × 10^+00^**
	Std	2.65 × 10^+00^	8.09 × 10^+00^	4.66 × 10^+00^	1.66 × 10^+00^	4.86 × 10^+00^	3.33 × 10^+00^	7.74 × 10^+00^	1.41 × 10^+01^	0.00 × 10^+00^
	C	+	+	+	+	+	+	+	+	/
F7	Mean	**0.00 × 10^+00^**	6.70 × 10^−02^	1.89 × 10^+00^	**0.00 × 10^+00^**	1.96 × 10^−01^	**0.00 × 10^+00^**	8.92 × 10^−01^	**0.00 × 10^+00^**	**0.00 × 10^+00^**
	Std	0.00 × 10^+00^	1.39 × 10^−02^	1.40 × 10^−01^	0.00 × 10^+00^	1.10 × 10^−01^	0.00 × 10^+00^	1.14 × 10^−01^	0.00 × 10^+00^	0.00 × 10^+00^
	C	=	+	+	=	+	=	+	=	/
F8	Mean	5.34 × 10^+00^	9.98 × 10^+00^	9.98 × 10^+00^	3.36 × 10^+00^	8.27 × 10^+00^	1.13 × 10^+01^	8.64 × 10^+00^	4.27 × 10^+00^	**3.43 × 10^−01^**
	Std	1.26 × 10^+00^	9.82 × 10^−01^	3.99 × 10^−01^	1.11 × 10^+00^	1.93 × 10^+00^	2.69 × 10^−01^	2.43 × 10^−01^	2.84 × 10^+00^	2.39 × 10^−01^
	C	+	+	+	+	+	+	+	+	/
F9	Mean	2.99 × 10^+00^	6.43 × 10^+00^	1.15 × 10^+01^	2.18 × 10^+00^	3.35 × 10^+00^	8.53 × 10^+00^	1.95 × 10^+01^	5.77 × 10^+00^	**9.93 × 10^−01^**
	Std	7.01 × 10^−01^	1.41 × 10^+00^	9.73 × 10^−01^	6.75 × 10^−01^	8.39 × 10^−01^	2.92 × 10^+00^	2.29 × 10^+00^	1.23 × 10^+00^	1.53 × 10^−01^
	C	+	+	+	+	+	+	+	+	/
F10	Mean	2.62 × 10^+00^	4.77 × 10^+02^	1.15 × 10^+03^	6.10 × 10^+00^	1.98 × 10^+01^	2.22 × 10^+00^	1.78 × 10^+03^	**4.30 × 10^−26^**	7.30 × 10^−09^
	Std	1.74 × 10^+00^	3.15 × 10^+02^	2.59 × 10^+02^	2.75 × 10^+00^	5.26 × 10^+00^	5.07 × 10^−01^	9.43 × 10^+01^	4.13 × 10^−26^	4.83 × 10^−09^
	C	+	+	+	+	+	+	+	-	/
F11	Mean	1.29 × 10^+01^	1.98 × 10^+01^	2.51 × 10^+01^	5.05 × 10^+01^	3.22 × 10^+01^	2.06 × 10^+00^	2.37 × 10^+01^	2.15 × 10^+01^	**1.19 × 10^−01^**
	Std	3.64 × 10^+00^	1.24 × 10^+00^	3.49 × 10^−01^	1.61 × 10^+01^	1.84 × 10^+01^	1.06 × 10^+00^	7.37 × 10^+00^	1.82 × 10^+01^	6.20 × 10^−02^
	C	+	+	+	+	+	+	+	+	/
F12	Mean	5.42 × 10^+00^	6.47 × 10^+00^	1.46 × 10^+01^	5.28 × 10^+00^	1.20 × 10^+01^	9.28 × 10^+00^	2.00 × 10^+01^	1.10 × 10^+01^	**2.84 × 10^+00^**
	Std	9.36 × 10^−01^	4.13 × 10^−01^	5.10 × 10^−01^	6.47 × 10^−01^	2.18 × 10^+00^	7.55 × 10^−01^	1.22 × 10^+00^	9.25 × 10^−01^	3.91 × 10^−01^
	C	+	+	+	+	+	+	+	+	/
F13	Mean	9.38 × 10^+00^	1.14 × 10^+01^	1.13 × 10^+01^	1.01 × 10^+01^	1.02 × 10^+01^	1.21 × 10^+01^	8.76 × 10^+00^	1.68 × 10^+01^	**7.71 × 10^+00^**
	Std	5.11 × 10^−01^	1.20 × 10^−01^	2.99 × 10^−01^	3.96 × 10^−01^	7.00 × 10^−01^	1.54 × 10^−01^	3.22 × 10^−01^	2.22 × 10^+00^	5.43 × 10^−01^
	C	+	+	+	+	+	+	+	+	/
F14	Mean	2.13 × 10^+01^	8.88 × 10^+01^	6.23 × 10^+01^	3.05 × 10^+01^	1.29 × 10^+02^	7.82 × 10^+01^	9.59 × 10^+01^	7.76 × 10^+01^	**9.09 × 10^−14^**
	Std	2.10 × 10^+00^	4.66 × 10^+00^	4.09 × 10^+00^	4.22 × 10^+00^	2.74 × 10^+01^	6.16 × 10^+00^	1.27 × 10^+01^	2.36 × 10^+01^	2.54 × 10^−14^
	C	+	+	+	+	+	+	+	+	/
F15	Mean	3.07 × 10^+00^	7.44 × 10^+00^	1.23 × 10^+01^	4.61 × 10^+00^	1.23 × 10^+01^	1.37 × 10^+01^	2.10 × 10^+01^	7.55 × 10^+00^	**1.09 × 10^+00^**
	Std	2.63 × 10^−01^	6.44 × 10^−01^	5.58 × 10^−01^	1.95 × 10^+00^	8.38 × 10^+00^	2.09 × 10^+00^	1.69 × 10^+00^	6.22 × 10^−01^	1.21 × 10^−01^
	C	+	+	+	+	+	+	+	+	/
F16	Mean	7.15 × 10^+00^	1.08 × 10^+01^	1.04 × 10^+01^	3.69 × 10^+00^	1.08 × 10^+01^	1.20 × 10^+01^	1.22 × 10^+01^	3.70 × 10^+00^	**2.91 × 10^−01^**
	Std	5.17 × 10^−01^	2.38 × 10^−01^	1.36 × 10^−01^	6.79 × 10^−01^	7.00 × 10^−01^	9.60 × 10^−02^	1.15 × 10^−01^	7.70 × 10^−01^	2.65 × 10^−12^
	C	+	+	+	+	+	+	+	+	/
F17	Mean	2.89 × 10^+08^	1.39 × 10^+04^	1.60 × 10^+03^	4.79 × 10^+08^	1.11 × 10^+09^	6.83 × 10^+03^	6.98 × 10^+07^	**6.11 × 10^−12^**	1.88 × 10^−02^
	Std	1.62 × 10^+08^	6.35 × 10^+03^	5.97 × 10^+02^	2.14 × 10^+08^	5.35 × 10^+08^	3.55 × 10^+03^	1.19 × 10^+07^	6.16 × 10^−12^	7.65 × 10^−03^
	C	+	+	+	+	+	+	+	-	/
F18	Mean	6.14 × 10^+00^	5.65 × 10^+02^	1.63 × 10^+03^	1.78 × 10^+03^	2.08 × 10^+04^	1.50 × 10^+01^	2.13 × 10^+04^	**6.82 × 10^−13^**	3.55 × 10^−07^
	Std	3.43 × 10^+00^	4.07 × 10^+02^	1.50 × 10^+02^	1.12 × 10^+03^	1.15 × 10^+04^	2.81 × 10^+00^	1.87 × 10^+03^	3.38 × 10^−13^	1.75 × 10^−07^
	C	+	+	+	+	+	+	+	-	/
F19	Mean	1.05 × 10^+01^	1.18 × 10^+01^	1.18 × 10^+01^	1.02 × 10^+01^	1.22 × 10^+01^	1.21 × 10^+01^	1.24 × 10^+01^	1.15 × 10^+01^	**1.01 × 10^+01^**
	Std	2.05 × 10^−01^	2.31 × 10^−01^	8.86 × 10^−02^	3.08 × 10^−01^	5.40 × 10^−01^	1.25 × 10^−01^	1.04 × 10^−01^	3.52 × 10^−01^	3.28 × 10^−01^
	C	+	+	+	=	+	+	+	+	/
F20	Mean	7.67 × 10^+00^	7.22 × 10^+00^	1.33 × 10^+01^	5.70 × 10^+00^	1.56 × 10^+01^	1.65 × 10^+01^	2.38 × 10^+01^	4.77 × 10^+00^	**4.18 × 10^+00^**
	Std	1.93 × 10^+00^	1.10 × 10^+00^	7.55 × 10^−01^	1.01 × 10^+00^	5.62 × 10^+00^	2.66 × 10^+00^	2.29 × 10^+00^	3.01 × 10^−01^	4.65 × 10^−01^
	C	+	+	+	+	+	+	+	+	/
	**W/T/L**	**18/1/1**	**19/0/1**	B	**18/2/0**	**20/0/0**	**19/1/0**	**20/0/0**	**13/1/6**	**/**

Note: the gray bacground highlights the best result on each function.

**Table 4:** Statistical results on 50 dimensions.

No.	Item	FDR	UPSO	FIPS	CLPSO	MPSO	FPA	SSO	dg-PSO	DG-PSO
F1	Mean	6.98 × 10^−08^	2.52 × 10^−05^	3.22 × 10^+03^	1.27 × 10^+02^	7.10 × 10^+00^	4.24 × 10^−01^	1.13 × 10^−02^	**7.35 × 10^−22^**	6.46 × 10^−18^
	Std	3.53 × 10^−08^	1.22 × 10^−05^	8.23 × 10^+02^	5.25 × 10^+01^	5.24 × 10^+00^	2.32 × 10^−01^	6.12 × 10^−03^	7.50 × 10^−22^	2.54 × 10^−18^
	C	+	+	+	+	+	+	+	-	/
F2	Mean	4.28 × 10^−164^	5.86 × 10^−200^	2.00 × 10^−22^	8.77 × 10^−74^	6.04 × 10^−13^	6.44 × 10^−05^	1.50 × 10^+03^	**6.55 × 10^−216^**	1.44 × 10^−121^
	Std	0.00 × 10^+00^	0.00 × 10^+00^	5.27 × 10^−23^	6.05 × 10^−74^	2.97 × 10^−13^	3.84 × 10^−05^	1.85 × 10^+02^	3.63 × 10^−216^	9.20 × 10^−122^
	C	-	-	+	+	+	+	+	+	/
F3	Mean	1.31 × 10^+03^	5.27 × 10^+03^	7.77 × 10^+03^	7.57 × 10^+02^	4.11 × 10^+03^	4.81 × 10^+03^	3.38 × 10^+03^	3.68 × 10^+03^	**4.50 × 10^+02^**
	Std	4.86 × 10^+02^	4.04 × 10^+02^	2.29 × 10^+02^	2.90 × 10^+02^	6.46 × 10^+02^	1.55 × 10^+02^	5.58 × 10^+02^	1.88 × 10^+03^	1.21 × 10^+02^
	C	+	+	+	+	+	+	+	+	/
F4	Mean	5.10 × 10^+03^	1.66 × 10^+04^	1.05 × 10^+04^	1.25 × 10^+04^	4.37 × 10^+02^	3.76 × 10^+02^	5.04 × 10^+03^	**8.21 × 10^+01^**	1.45 × 10^+02^
	Std	3.72 × 10^+03^	4.01 × 10^+03^	1.34 × 10^+03^	5.04 × 10^+03^	1.74 × 10^+02^	1.09 × 10^+02^	2.49 × 10^+03^	3.57 × 10^+01^	7.05 × 10^+01^
	C	+	+	+	+	+	+	+	-	/
F5	Mean	2.49 × 10^+01^	1.92 × 10^−01^	4.22 × 10^+01^	9.21 × 10^+01^	7.25 × 10^−02^	9.03 × 10^+00^	3.79 × 10^+01^	1.85 × 10^+00^	**7.21 × 10^−02^**
	Std	1.27 × 10^+01^	9.25 × 10^−02^	1.96 × 10^−01^	2.78 × 10^+01^	4.75 × 10^−02^	5.00 × 10^+00^	3.11 × 10^+00^	1.72 × 10^+00^	4.46 × 10^−01^
	C	+	+	+	+	=	+	+	+	/
F6	Mean	6.29 × 10^+01^	1.31 × 10^+02^	1.67 × 10^+02^	1.43 × 10^+01^	7.02 × 10^+01^	6.94 × 10^+01^	6.95 × 10^+01^	5.94 × 10^+01^	**6.04 × 10^−15^**
	Std	6.33 × 10^+00^	1.72 × 10^+01^	9.78 × 10^+00^	5.67 × 10^−01^	1.65 × 10^+01^	8.82 × 10^+00^	7.57 × 10^+00^	1.48 × 10^+01^	7.94 × 10^−16^
	C	+	+	+	+	+	+	+	+	/
F7	Mean	**0.00 × 10^+00^**	1.45 × 10^−01^	2.60 × 10^+00^	**0.00 × 10^+00^**	3.05 × 10^−01^	**0.00 × 10^+00^**	1.46 × 10^+00^	**0.00 × 10^+00^**	**0.00 × 10^+00^**
	Std	0.00 × 10^+00^	1.28 × 10^−02^	9.52 × 10^−02^	0.00 × 10^+00^	1.75 × 10^−01^	0.00 × 10^+00^	1.25 × 10^−01^	0.00 × 10^+00^	0.00 × 10^+00^
	C	=	+	+	=	+	=	+	+	/
F8	Mean	1.16 × 10^+01^	2.00 × 10^+01^	1.96 × 10^+01^	4.93 × 10^+00^	1.98 × 10^+01^	1.91 × 10^+01^	1.54 × 10^+01^	1.05 × 10^+01^	**8.74 × 10^−01^**
	Std	2.37 × 10^+00^	4.87 × 10^−01^	5.52 × 10^−01^	1.73 × 10^+00^	7.46 × 10^−01^	9.88 × 10^−01^	5.30 × 10^−01^	4.85 × 10^−01^	1.76 × 10^−01^
	C	+	+	+	+	+	+	+	+	/
F9	Mean	7.66 × 10^+00^	1.58 × 10^+01^	2.83 × 10^+01^	3.85 × 10^+00^	1.08 × 10^+01^	2.21 × 10^+01^	3.53 × 10^+01^	6.36 × 10^+00^	**1.88 × 10^+00^**
	Std	1.16 × 10^+00^	3.02 × 10^+00^	1.05 × 10^+00^	9.01 × 10^−01^	2.79 × 10^+00^	3.53 × 10^+00^	2.68 × 10^+00^	9.90 × 10^−01^	3.46 × 10^−01^
	C	+	+	+	+	+	+	+	+	/
F10	Mean	1.02 × 10^+02^	1.46 × 10^+03^	8.12 × 10^+03^	4.46 × 10^+02^	2.81 × 10^+02^	1.14 × 10^+02^	3.08 × 10^+03^	**2.69 × 10^−07^**	9.16 × 10^−05^
	Std	1.68 × 10^+01^	4.09 × 10^+02^	7.63 × 10^+02^	7.17 × 10^+01^	1.01 × 10^+02^	5.14 × 10^+01^	1.87 × 10^+02^	2.24 × 10^−07^	3.04 × 10^−05^
	C	+	+	+	+	+	+	+	+	/
F11	Mean	6.08 × 10^+01^	5.25 × 10^+01^	4.49 × 10^+01^	1.16 × 10^+02^	5.98 × 10^+01^	4.61 × 10^−02^	5.53 × 10^+01^	1.70 × 10^+01^	**2.02 × 10^−02^**
	Std	1.35 × 10^+01^	1.27 × 10^+01^	3.52 × 10^−01^	4.43 × 10^+01^	1.63 × 10^+01^	1.62 × 10^−02^	7.07 × 10^+00^	1.32 × 10^+01^	1.05 × 10^−02^
	C	+	+	+	+	+	+	+	+	/
F12	Mean	1.15 × 10^+01^	2.42 × 10^+01^	3.13 × 10^+01^	1.19 × 10^+01^	2.38 × 10^+01^	2.19 × 10^+01^	4.23 × 10^+01^	**1.78 × 10^+00^**	8.18 × 10^+00^
	Std	1.33 × 10^+00^	3.59 × 10^+00^	2.74 × 10^−01^	1.09 × 10^+00^	2.57 × 10^+00^	8.40 × 10^−01^	3.23 × 10^+00^	6.58 × 10^−01^	6.69 × 10^−01^
	C	+	+	+	+	+	+	+	-	/
F13	Mean	1.73 × 10^+01^	2.03 × 10^+01^	2.13 × 10^+01^	1.89 × 10^+01^	1.87 × 10^+01^	2.11 × 10^+01^	1.60 × 10^+01^	2.62 × 10^+01^	**1.58 × 10^+01^**
	Std	5.78 × 10^−01^	4.40 × 10^−01^	1.64 × 10^−01^	3.40 × 10^−01^	7.67 × 10^−01^	1.84 × 10^−01^	5.61 × 10^−01^	6.18 × 10^+00^	5.60 × 10^−01^
	C	+	+	+	+	+	+	=	+	/
F14	Mean	7.73 × 10^+01^	1.93 × 10^+02^	1.73 × 10^+02^	6.77 × 10^+01^	2.82 × 10^+02^	1.55 × 10^+02^	2.83 × 10^+02^	2.48 × 10^+02^	**1.59 × 10^−13^**
	Std	7.70 × 10^+00^	1.76 × 10^+01^	1.20 × 10^+01^	4.42 × 10^+00^	3.04 × 10^+01^	1.07 × 10^+01^	2.23 × 10^+01^	2.04 × 10^+01^	3.11 × 10^−14^
	C	+	+	+	+	+	+	+	+	/
F15	Mean	2.50 × 10^+01^	2.16 × 10^+01^	2.71 × 10^+01^	5.84 × 10^+03^	1.07 × 10^+01^	5.38 × 10^+01^	4.95 × 10^+01^	2.14 × 10^+01^	**1.62 × 10^+00^**
	Std	1.03 × 10^+01^	3.16 × 10^+00^	9.78 × 10^−01^	4.23 × 10^+03^	1.73 × 10^+00^	9.85 × 10^+00^	1.06 × 10^+00^	3.81 × 10^−01^	7.70 × 10^−02^
	C	+	+	+	+	+	+	+	+	/
F16	Mean	1.26 × 10^+01^	2.02 × 10^+01^	2.03 × 10^+01^	5.43 × 10^+00^	2.09 × 10^+01^	2.10 × 10^+01^	2.17 × 10^+01^	1.40 × 10^+01^	**8.70 × 10^−01^**
	Std	1.01 × 10^+00^	2.30 × 10^−01^	6.67 × 10^−02^	5.09 × 10^−01^	9.02 × 10^−01^	2.92 × 10^−01^	8.77 × 10^−02^	5.39 × 10^+00^	2.86 × 10^−01^
	C	+	+	+	+	+	+	+	+	/
F17	Mean	1.41 × 10^+09^	**1.96 × 10^+03^**	4.83 × 10^+04^	3.83 × 10^+09^	2.03 × 10^+10^	1.51 × 10^+04^	2.89 × 10^+08^	7.71 × 10^+08^	1.58 × 10^+04^
	Std	7.06 × 10^+08^	9.96 × 10^+02^	3.28 × 10^+04^	1.15 × 10^+09^	1.38 × 10^+10^	5.62 × 10^+03^	6.63 × 10^+07^	8.60 × 10^+08^	8.68 × 10^+03^
	C	+	-	+	+	+	=	+	+	/
F18	Mean	1.88 × 10^+03^	4.90 × 10^+03^	1.09 × 10^+04^	3.05 × 10^+03^	3.73 × 10^+03^	1.48 × 10^+03^	6.35 × 10^+04^	**6.23 × 10^−09^**	3.37 × 10^−03^
	Std	1.28 × 10^+03^	5.95 × 10^+02^	9.56 × 10^+02^	1.00 × 10^+03^	1.57 × 10^+03^	3.26 × 10^+02^	3.80 × 10^+03^	2.11 × 10^−09^	1.52 × 10^−03^
	C	+	+	+	+	+	+	+	-	/
F19	Mean	1.95 × 10^+01^	2.12 × 10^+01^	2.17 × 10^+01^	**1.92 × 10^+01^**	2.07 × 10^+01^	2.17 × 10^+01^	2.22 × 10^+01^	2.07 × 10^+01^	1.93 × 10^+01^
	Std	5.51 × 10^−01^	1.08 × 10^−01^	1.44 × 10^−01^	3.62 × 10^−01^	1.55 × 10^−01^	2.64 × 10^−01^	6.31 × 10^−02^	8.82 × 10^−01^	2.37 × 10^−01^
	C	=	+	+	=	+	+	+	+	
F20	Mean	7.71 × 10^+01^	2.70 × 10^+01^	3.19 × 10^+01^	1.15 × 10^+02^	3.94 × 10^+02^	4.45 × 10^+01^	5.55 × 10^+01^	1.19 × 10^+01^	**8.64 × 10^+00^**
	Std	4.93 × 10^+01^	4.94 × 10^+00^	6.65 × 10^−01^	5.86 × 10^+01^	1.99 × 10^+02^	7.92 × 10^+00^	4.90 × 10^+00^	7.90 × 10^+00^	9.42 × 10^−01^
	C	+	+	+	+	+	+	+	+	/
	**W/T/L**	**17/2/1**	**19/0/1**	**20/0/0**	**19/1/0**	**19/1/0**	**19/2/0**	**19/1/0**	**13/1/6**	**/**

Note: the gray bacground highlights the best result on each function.

**Table 5:** Numerical rankings of the Friedman test.

Dimensions	FDR	UPSO	FIPS	CLPSO	MPSO	FPA	SSO	dg-PSO	DG-PSO
30-*D*	3.45	5.625	6.725	4.2	6.95	5.9	7.3	3.35	**1.5**
50-*D*	4.25	5.6	7.075	5	6.025	5.275	6.85	3.275	**1.65**

**Table 6:** Parameters and references of the algorithms.

Algorithm	Parameters	Value	Reference
DE	Population size	40	[62]
	Scaling factor	0.8	
	Crossover possibility	0.25	
ABC	Swam size	20	[59]
	Max trial limit	50	
CS	Number of nests	25	[53]
	Step size	1	
	Mutation probability value	0.25	
	Scale factor	1.5	
MPSO	Maximum, minimum swarm size	40, 5	[36]
	acceleration constants $c_{1},\begin{array}{c} c_{2} \end{array}$	2, 2	
	Maximum, minimum inertial weight	0.9, 0.3	
SSO	Population size	50	[47]
	The threshold PF	0.7	

**Table 7:** Statistical results.

Image	*D*	Item	DE	ABC	CS	MPSO	SSO	Ours
a	2	Mean	**1.79165 × 10^+03^**	**1.79165 × 10^+03^**	**1.79165 × 10^+03^**	**1.79165 × 10^+03^**	**1.79165 × 10^+03^**	**1.79165 × 10^+03^**
		Std	4.67 × 10^−13^	4.67 × 10^−13^	4.67 × 10^−13^	4.67 × 10^−13^	4.67 × 10^−13^	4.67 × 10^−13^
	3	Mean	1.94917 × 10^+03^	1.94918 × 10^+03^	**1.94919 × 10^+03^**	**1.94919 × 10^+03^**	**1.94919 × 10^+03^**	**1.94919 × 10^+03^**
		Std	6.90 × 10^−02^	1.59 × 10^−02^	9.33 × 10^−13^	9.33 × 10^−13^	9.33 × 10^−13^	9.33 × 10^−13^
	4	Mean	2.03403 × 10^+03^	2.03416 × 10^+03^	**2.03427 × 10^+03^**	**2.03427 × 10^+03^**	**2.03427 × 10^+03^**	**2.03427 × 10^+03^**
		Std	6.01 × 10^−01^	5.45 × 10^−02^	0.00 × 10^+00^	0.00 × 10^+00^	0.00 × 10^+00^	0.00 × 10^+00^
	5	Mean	2.06635 × 10^+03^	2.06618 × 10^+03^	**2.06648 × 10^+03^**	**2.06648 × 10^+03^**	**2.06648 × 10^+03^**	**2.06648 × 10^+03^**
		Std	2.74 × 10^−01^	1.79 × 10^−01^	4.50 × 10^−03^	5.84 × 10^−03^	0.00 × 10^+00^	0.00 × 10^+00^
	7	Mean	2.09715 × 10^+03^	2.09653 × 10^+03^	2.09734 × 10^+03^	**2.09739 × 10^+03^**	**2.09739 × 10^+03^**	**2.09739 × 10^+03^**
		Std	2.25 × 10^−01^	4.38 × 10^−01^	4.63 × 10^−02^	4.20 × 10^−04^	1.42 × 10^−03^	4.67 × 10^−13^
	9	Mean	2.11153 × 10^+03^	2.11097 × 10^+03^	2.11188 × 10^+03^	2.11158 × 10^+03^	**2.11192 × 10^+03^**	**2.11192 × 10^+03^**
		Std	4.20 × 10^−01^	3.52 × 10^−01^	6.55 × 10^−02^	2.43 × 10^−01^	8.36 × 10^−02^	9.06 × 10^−02^
	15	Mean	2.12912 × 10^+03^	2.12857 × 10^+03^	2.12965 × 10^+03^	2.12976 × 10^+03^	2.12922 × 10^+03^	**2.12995 × 10^+03^**
		Std	8.17 × 10^−01^	2.30 × 10^−01^	1.69 × 10^−01^	3.25 × 10^−01^	6.58 × 10^−01^	1.40 × 10^−01^
	20	Mean	2.13280 × 10^+03^	2.13370 × 10^+03^	2.13444 × 10^+03^	2.13464 × 10^+03^	2.13333 × 10^+03^	**2.13472 × 10^+03^**
		Std	7.99 × 10^−01^	2.01 × 10^−01^	1.18 × 10^−01^	5.27 × 10^−01^	6.86 × 10^−01^	1.44 × 10^−01^
b	2	Mean	**2.27112 × 10^+03^**	**2.27112 × 10^+03^**	**2.27112 × 10^+03^**	**2.27112 × 10^+03^**	**2.27112 × 10^+03^**	**2.27112 × 10^+03^**
		Std	9.33 × 10^−13^	9.33 × 10^−13^	9.33 × 10^−13^	9.33 × 10^−13^	9.33 × 10^−13^	9.33 × 10^−13^
	3	Mean	2.50343 × 10^+03^	2.50346 × 10^+03^	**2.50347 × 10^+03^**	**2.50347 × 10^+03^**	**2.50347 × 10^+03^**	**2.50347 × 10^+03^**
		Std	1.37 × 10^−01^	1.40 × 10^−02^	4.67 × 10^−13^	4.67 × 10^−13^	4.67 × 10^−13^	4.67 × 10^−13^
	4	Mean	2.58706 × 10^+03^	2.58695 × 10^+03^	**2.58710 × 10^+03^**	2.58709 × 10^+03^	**2.58710 × 10^+03^**	**2.58710 × 10^+03^**
		Std	1.12 × 10^−01^	1.30 × 10^−01^	4.67 × 10^−13^	4.67 × 10^−13^	4.67 × 10^−13^	4.67 × 10^−13^
	5	Mean	2.62673 × 10^+03^	2.62643 × 10^+03^	2.62685 × 10^+03^	**2.62686 × 10^+03^**	**2.62686 × 10^+03^**	**2.62686 × 10^+03^**
		Std	1.49 × 10^−01^	2.85 × 10^−01^	1.39 × 10^−02^	6.24 × 10^−02^	9.33 × 10^−13^	9.33 × 10^−13^
	7	Mean	2.66456 × 10^+03^	2.66401 × 10^+03^	2.66481 × 10^+03^	2.66488 × 10^+03^	**2.66490 × 10^+03^**	**2.66490 × 10^+03^**
		Std	2.78 × 10^−01^	2.76 × 10^−01^	5.67 × 10^−02^	2.08 × 10^−01^	4.67 × 10^−13^	4.67 × 10^−13^
	9	Mean	2.68400 × 10^+03^	2.68338 × 10^+03^	2.68448 × 10^+03^	2.68458 × 10^+03^	2.68457 × 10^+03^	**2.68459 × 10^+03^**
		Std	4.71 × 10^−01^	4.59 × 10^−01^	5.68 × 10^−02^	3.55 × 10^−01^	2.69 × 10^−02^	8.48 × 10^−03^
	15	Mean	2.70493 × 10^+03^	2.70461 × 10^+03^	2.70564 × 10^+03^	2.70564 × 10^+03^	2.70535 × 10^+03^	**2.70588 × 10^+03^**
		Std	7.97 × 10^−01^	2.72 × 10^−01^	1.34 × 10^−01^	3.15 × 10^−01^	1.10 × 10^+00^	7.63 × 10^−02^
	20	Mean	2.70907 × 10^+03^	2.71046 × 10^+03^	2.71127 × 10^+03^	2.71168 × 10^+03^	2.71019 × 10^+03^	**2.71153 × 10^+03^**
		Std	1.30 × 10^+00^	2.09 × 10^−01^	1.54 × 10^−01^	5.25 × 10^−01^	7.90 × 10^−01^	2.79 × 10^−01^
c	2	Mean	**2.67669 × 10^+03^**	**2.67669 × 10^+03^**	**2.67669 × 10^+03^**	**2.67669 × 10^+03^**	**2.67669 × 10^+03^**	**2.67669 × 10^+03^**
		Std	9.33 × 10^−13^	9.33 × 10^−13^	9.33 × 10^−13^	9.33 × 10^−13^	9.33 × 10^−13^	9.33 × 10^−13^
	3	Mean	2.87034 × 10^+03^	2.87040 × 10^+03^	**2.87042 × 10^+03^**	**2.87042 × 10^+03^**	**2.87042 × 10^+03^**	**2.87042 × 10^+03^**
		Std	2.80 × 10^−01^	2.94 × 10^−02^	9.33 × 10^−13^	9.33 × 10^−13^	9.33 × 10^−13^	9.33 × 10^−13^
	4	Mean	2.93107 × 10^+03^	2.93110 × 10^+03^	2.93128 × 10^+03^	**2.93129 × 10^+03^**	**2.93129 × 10^+03^**	**2.93129 × 10^+03^**
		Std	5.24 × 10^−01^	1.69 × 10^−01^	2.87 × 10^−03^	2.36 × 10^−01^	1.40 × 10^−12^	1.40 × 10^−12^
	5	Mean	2.96630 × 10^+03^	2.96622 × 10^+03^	**2.96645 × 10^+03^**	**2.96645 × 10^+03^**	**2.96645 × 10^+03^**	**2.96645 × 10^+03^**
		Std	3.08 × 10^−01^	1.72 × 10^−01^	1.90 × 10^−03^	1.65 × 10^−01^	1.40 × 10^−12^	1.57 × 10^−03^
	7	Mean	2.99969 × 10^+03^	2.99923 × 10^+03^	**2.99982 × 10^+03^**	2.99979 × 10^+03^	2.99980 × 10^+03^	2.99981 × 10^+03^
		Std	2.09 × 10^−01^	2.68 × 10^−01^	4.74 × 10^−02^	9.48 × 10^−03^	8.60 × 10^−02^	8.25 × 10^−02^
	9	Mean	3.01430 × 10^+03^	3.01377 × 10^+03^	3.01450 × 10^+03^	3.01462 × 10^+03^	3.01467 × 10^+03^	**3.01474 × 10^+03^**
		Std	4.31 × 10^−01^	3.59 × 10^−01^	2.06 × 10^−01^	3.49 × 10^−01^	3.39 × 10^−01^	2.45 × 10^−01^
	15	Mean	3.02911 × 10^+03^	3.02886 × 10^+03^	3.02963 × 10^+03^	3.02973 × 10^+03^	3.02936 × 10^+03^	**3.02989 × 10^+03^**
		Std	8.23 × 10^−01^	1.91 × 10^−01^	1.53 × 10^−01^	2.02 × 10^−01^	8.09 × 10^−01^	2.10 × 10^−01^
	20	Mean	3.03250 × 10^+03^	3.03354 × 10^+03^	3.03409 × 10^+03^	3.03443 × 10^+03^	3.03310 × 10^+03^	**3.03447 × 10^+03^**
		Std	7.16 × 10^−01^	1.50 × 10^−01^	1.07 × 10^−01^	5.52 × 10^−01^	6.31 × 10^−01^	1.05 × 10^−01^
d	2	Mean	**3.50826 × 10^+03^**	**3.50826 × 10^+03^**	**3.50826 × 10^+03^**	**3.50826 × 10^+03^**	**3.50826 × 10^+03^**	**3.50826 × 10^+03^**
		Std	1.40 × 10^−12^	1.40 × 10^−12^	1.40 × 10^−12^	1.40 × 10^−12^	1.40 × 10^−12^	1.40 × 10^−12^
	3	Mean	3.65657 × 10^+03^	3.65661 × 10^+03^	**3.65665 × 10^+03^**	**3.65665 × 10^+03^**	**3.65665 × 10^+03^**	**3.65665 × 10^+03^**
		Std	2.31 × 10^−01^	5.15 × 10^−02^	2.33 × 10^−12^	2.05 × 10^−02^	2.33 × 10^−12^	2.33 × 10^−12^
	4	Mean	3.73546 × 10^+03^	3.73537 × 10^+03^	**3.73562 × 10^+03^**	**3.73562 × 10^+03^**	**3.73562 × 10^+03^**	**3.73562 × 10^+03^**
		Std	2.28 × 10^−01^	1.56 × 10^−01^	4.67 × 10^−13^	2.78 × 10^−02^	4.67 × 10^−13^	4.67 × 10^−13^
	5	Mean	3.78074 × 10^+03^	3.78052 × 10^+03^	**3.78100 × 10^+03^**	3.78099 × 10^+03^	**3.78100 × 10^+03^**	**3.78100 × 10^+03^**
		Std	4.10 × 10^−01^	2.56 × 10^−01^	1.23 × 10^−02^	3.15 × 10^−01^	9.33 × 10^−13^	9.33 × 10^−13^
	7	Mean	3.81857 × 10^+03^	3.81818 × 10^+03^	**3.81897 × 10^+03^**	3.81864 × 10^+03^	3.81865 × 10^+03^	3.81883 × 10^+03^
		Std	6.02 × 10^−01^	4.10 × 10^−01^	3.38 × 10^−02^	4.35 × 10^−01^	1.11 × 10^+00^	8.17 × 10^−01^
	9	Mean	3.83628 × 10^+03^	3.83555 × 10^+03^	3.83665 × 10^+03^	3.83681 × 10^+03^	3.83680 × 10^+03^	**3.83682 × 10^+03^**
		Std	5.38 × 10^−01^	4.48 × 10^−01^	8.98 × 10^−02^	5.02 × 10^−01^	1.62 × 10^−02^	3.18 × 10^−03^
	15	Mean	3.85501 × 10^+03^	3.85465 × 10^+03^	3.85558 × 10^+03^	3.85598 × 10^+03^	3.85543 × 10^+03^	**3.85593 × 10^+03^**
		Std	9.03 × 10^−01^	2.48 × 10^−01^	1.44 × 10^−01^	8.12 × 10^−01^	6.33 × 10^−01^	1.62 × 10^−01^
	20	Mean	3.85891 × 10^+03^	3.86036 × 10^+03^	3.86097 × 10^+03^	3.86131 × 10^+03^	3.86011 × 10^+03^	**3.86135 × 10^+03^**
		Std	9.91 × 10^−01^	2.52 × 10^−01^	1.64 × 10^−01^	6.12 × 10^−01^	7.65 × 10^−01^	1.97 × 10^−01^
e	2	Mean	**1.07218 × 10^+03^**	**1.07218 × 10^+03^**	**1.07218 × 10^+03^**	**1.07218 × 10^+03^**	**1.07218 × 10^+03^**	**1.07218 × 10^+03^**
		Std	0.00 × 10^+00^	0.00 × 10^+00^	0.00 × 10^+00^	0.00 × 10^+00^	0.00 × 10^+00^	0.00 × 10^+00^
	3	Mean	1.17024 × 10^+03^	1.17021 × 10^+03^	**1.17025 × 10^+03^**	1.17024 × 10^+03^	**1.17025 × 10^+03^**	**1.17025 × 10^+03^**
		Std	1.49 × 10^−02^	4.97 × 10^−02^	2.33 × 10^−13^	2.33 × 10^−13^	2.33 × 10^−13^	2.33 × 10^−13^
	4	Mean	1.21391 × 10^+03^	1.21383 × 10^+03^	1.21398 × 10^+03^	**1.21399 × 10^+03^**	**1.21399 × 10^+03^**	**1.21399 × 10^+03^**
		Std	1.29 × 10^−01^	1.23 × 10^−01^	6.60 × 10^−03^	3.89 × 10^−02^	2.33 × 10^−13^	2.33 × 10^−13^
	5	Mean	1.24246 × 10^+03^	1.24221 × 10^+03^	1.24261 × 10^+03^	**1.24265 × 10^+03^**	**1.24265 × 10^+03^**	**1.24265 × 10^+03^**
		Std	3.18 × 10^−01^	1.81 × 10^−01^	6.27 × 10^−02^	1.95 × 10^−01^	4.67 × 10^−13^	4.67 × 10^−13^
	7	Mean	1.27623 × 10^+03^	1.27577 × 10^+03^	1.27656 × 10^+03^	1.27660 × 10^+03^	**1.27661 × 10^+03^**	**1.27661 × 10^+03^**
		Std	5.52 × 10^−01^	4.12 × 10^−01^	5.43 × 10^−02^	2.55 × 10^−01^	2.33 × 10^−13^	2.33 × 10^−13^
	9	Mean	1.29227 × 10^+03^	1.29152 × 10^+03^	1.29254 × 10^+03^	1.29267 × 10^+03^	1.29267 × 10^+03^	**1.29268 × 10^+03^**
		Std	4.55 × 10^−01^	4.68 × 10^−01^	7.29 × 10^−02^	3.04 × 10^−01^	1.42 × 10^−02^	3.65 × 10^−03^
	15	Mean	1.30947 × 10^+03^	1.30914 × 10^+03^	1.30986 × 10^+03^	1.31018 × 10^+03^	1.30972 × 10^+03^	**1.31035 × 10^+03^**
		Std	7.66 × 10^−01^	3.43 × 10^−01^	2.49 × 10^−01^	3.64 × 10^−01^	6.56 × 10^−01^	1.45 × 10^−01^
	20	Mean	1.31345 × 10^+03^	1.31412 × 10^+03^	1.31467 × 10^+03^	1.31501 × 10^+03^	1.31379 × 10^+03^	**1.31522 × 10^+03^**
		Std	8.91 × 10^−01^	2.70 × 10^−01^	1.94 × 10^−01^	4.25 × 10^−01^	7.16 × 10^−01^	1.09 × 10^−01^

Note: the gray bacground highlights the best result on each function.

**Table 8:** Numerical rankings of the Friedman tests.

Level	DE	ABC	CS	MPSO	SSO	Ours
Normal level	5.25	4.775	2.8625	3.3125	2.775	**2.025**
High level	5.3125	4.7625	2.6625	3.1125	3.2	**1.95**
